# First metatarsophalangeal joint range of motion is associated with lower limb kinematics in individuals with first metatarsophalangeal joint osteoarthritis

**DOI:** 10.1186/s13047-020-00404-0

**Published:** 2020-06-08

**Authors:** Jamie J. Allan, Jodie A. McClelland, Shannon E. Munteanu, Andrew K. Buldt, Karl B. Landorf, Edward Roddy, Maria Auhl, Hylton B. Menz

**Affiliations:** 1 Discipline of Podiatry, School of Allied Health, Human Services and Sport, La Trobe University, Melbourne, Victoria 3086 Australia; 2 La Trobe Sport and Exercise Medicine Research Centre, School of Allied Health, Human Services and Sport, La Trobe University, Melbourne, Victoria 3086 Australia; 3 Discipline of Physiotherapy, School of Allied Health, Human Services and Sport, La Trobe University, Melbourne, Victoria 3086 Australia; 4 Primary Care Centre Versus Arthritis, School of Primary, Community and Social Care, Keele University, Keele, Staffordshire ST5 5BG UK; 5 Haywood Academic Rheumatology Centre, Midlands Partnership NHS Foundation Trust, Haywood Hospital, Burslem, Staffordshire ST6 7AG UK

## Abstract

**Background:**

Osteoarthritis of the first metatarsophalangeal joint (1st MTP joint OA) is a common and disabling condition that results in pain and limited joint range of motion. There is inconsistent evidence regarding the relationship between clinical measurement of 1st MTP joint maximum dorsiflexion and dynamic function of the joint during level walking. Therefore, the aim of this study was to examine the association between passive non-weightbearing (NWB) 1st MTP joint maximum dorsiflexion and sagittal plane kinematics in individuals with radiographically confirmed 1st MTP joint OA.

**Methods:**

Forty-eight individuals with radiographically confirmed 1st MTP joint OA (24 males and 24 females; mean age 57.8 years, standard deviation 10.5) underwent clinical measurement of passive NWB 1st MTP joint maximum dorsiflexion and gait analysis during level walking using a 10-camera infrared Vicon motion analysis system. Sagittal plane kinematics of the 1st MTP, ankle, knee, and hip joints were calculated. Associations between passive NWB 1st MTP joint maximum dorsiflexion and kinematic variables were explored using Pearson’s *r* correlation coefficients.

**Results:**

Passive NWB 1st MTP joint maximum dorsiflexion was significantly associated with maximum 1st MTPJ dorsiflexion (*r =* 0.486, *p* < 0.001), ankle joint maximum plantarflexion (*r =* 0.383, *p =* 0.007), and ankle joint excursion (*r =* 0.399, *p =* 0.005) during gait. There were no significant associations between passive NWB 1st MTP joint maximum dorsiflexion and sagittal plane kinematics of the knee or hip joints.

**Conclusions:**

These findings suggest that clinical measurement of 1st MTP joint maximum dorsiflexion provides useful insights into the dynamic function of the foot and ankle during the propulsive phase of gait in this population.

## Background

Osteoarthritis of the first metatarsophalangeal joint (1st MTP joint OA) has been recognised as one of the most common causes of foot pain in middle-aged and older people [1]. The condition affects 8% of individuals aged over 50 years and leads to disability, poorer health-related quality of life, and impaired locomotor function [1]. 1st MTP joint OA is characterised by joint pain and stiffness, dorsal exostosis formation, and reduced 1st MTP joint dorsiflexion range of motion [2]. The presence of adequate 1st MTP joint dorsiflexion is essential during the terminal stance and pre-swing phases of gait to enable smooth forward progression of the body over the foot [3]. As a consequence of limited motion within the joint, individuals with 1st MTP joint OA adopt an altered gait pattern, characterised by reduced step length and shorter stance duration [4, 5].

Three studies have explored the relationship between clinical measurement of 1st MTP joint motion and dynamic function during walking, with inconsistent findings [3, 6, 7]. In pain-free, healthy individuals, Nawoczenski et al. [3] found significant associations between 1st MTP joint maximum dorsiflexion during walking and active weightbearing (Pearson’s *r* = 0.80), passive weightbearing (*r* = 0.61) and passive non-weightbearing (*r* = 0.67) 1st MTP joint ROM. Similarly, in asymptomatic individuals, Jarvis et al. [6] found a significant association (*r* = 0.32) between 1st MTP joint maximum dorsiflexion and maximal dorsiflexion during walking. In contrast, Halstead et al. [7] found no significant association between passive 1st MTP joint maximum dorsiflexion and 1st MTP joint maximum dorsiflexion during walking in individuals with limited 1st MTP joint motion (as determined by Jack’s test [8] in relaxed standing). To the best of our knowledge, no studies have examined this association in individuals with radiographically-confirmed 1st MTP joint OA.

Therefore, the primary aim of this study was to determine whether there is an association between passive non-weightbearing (NWB) 1st MTP joint maximum dorsiflexion and sagittal plane kinematics in individuals with radiographically confirmed 1st MTP joint OA. Doing so will provide insight into the underlying mechanisms responsible for gait alterations in individuals with this condition.

## Methods

### Participants

Participants for this study were drawn from a larger randomised trial evaluating the effectiveness of shoe-stiffening inserts for 1st MTP joint OA, the details of which have been published previously [9]. The La Trobe University Human Ethics Committee provided ethical approval (number HEC15–128) and all participants provided written informed consent prior to enrolment. Briefly, individuals with 1st MTP joint OA were recruited by advertisements placed in local newspapers, posters placed in senior citizens’ centres and retirement villages, mail-out advertisements to health-care practitioners in Melbourne, mail-outs to people currently accessing podiatry services at the La Trobe University Health Sciences Clinic, and through social networking media (e.g. Facebook, Twitter). Inclusion criteria were: (i) 18 years of age or older, (ii) pain in the 1st MTP joint on most days for at least 12 weeks, (iii) pain rated at least 30 mm on a 100 mm visual analogue scale (VAS), (iv) pain upon palpation of the dorsal aspect of the 1st MTP joint, (v) able to walk household distances (> 50 m) without the aid of a walker, crutches or cane, and (vi) willing to have their foot x-rayed. Exclusion criteria included: (i) previous first MTP joint surgery, (ii) currently pregnant, (iii) significant first MTP joint deformity including hallux valgus, (iv) presence of any systemic inflammatory condition such as gout or rheumatoid arthritis, (v) an inability to speak and read English, and (vi) cognitive impairment.

### Clinical and radiographic assessment

**Fig. 1:**
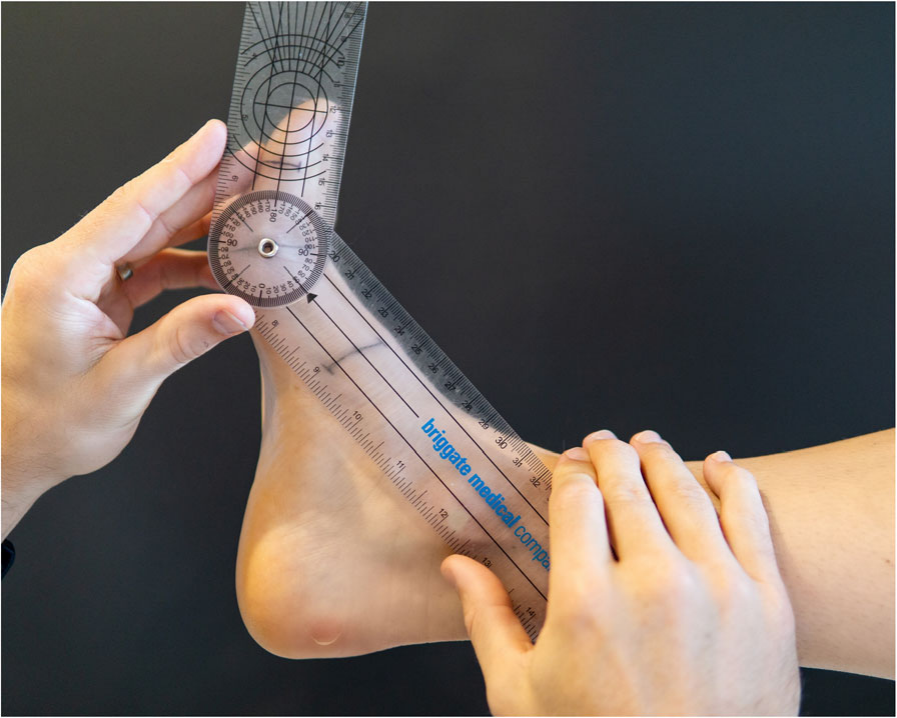
Measurement of passive NWB 1st MTP joint maximum dorsiflexion

Participant characteristics (such as age, sex, weight, height, education and income level), major medical conditions and number of medications were obtained via a structured questionnaire. Height and weight were measured using a stadiometer and digital scales, and body mass index (BMI) was calculated as weight (kg) / height (m)^2^. Static foot posture was assessed using the Foot Posture Index [10]. Passive NWB 1st MTP joint maximum dorsiflexion as measured using a reliable goniometric technique [11]. The first metatarsal and proximal phalanx of the hallux were bisected in the sagittal plane. A dorsiflexion force was applied to the hallux until end range of motion was reached, allowing the first ray to maximally plantarflex. The angle between the two lines was then measured via a handheld goniometer (see Fig. 1). The reliability of this test has been shown to be excellent in healthy individuals [12] and individuals with 1st MTP joint OA [11] (intra-class correlation coefficient = 0.95). Clinical features associated with 1st MTP joint OA (pain on palpation, dorsal exostosis, joint effusion, pain on motion, hard-end feel and crepitus) were documented [11]. The presence of radiographic 1st MTP joint OA was determined at baseline using the La Trobe University radiographic atlas [13]. The atlas incorporates weightbearing dorso-plantar and lateral radiographs to document the presence of OA based on observations of osteophytes and joint space narrowing. Osteophytes were recorded as absent (score = 0), small (score = 1), moderate (score = 2) or severe (score = 3). Joint space narrowing was recorded as none (score = 0), definite (score = 1), severe (score = 2) or joint fusion (score = 3). Radiographic OA using this atlas is defined as a score of 2 or more for osteophytes or joint space narrowing on either dorso-plantar and lateral views. The atlas has been shown to have good to excellent intra- and inter-rater reliability for grading 1st MTP joint OA (ĸ range 0.64 to 0.95) [13].

### Biomechanical assessment

**Fig. 2:**
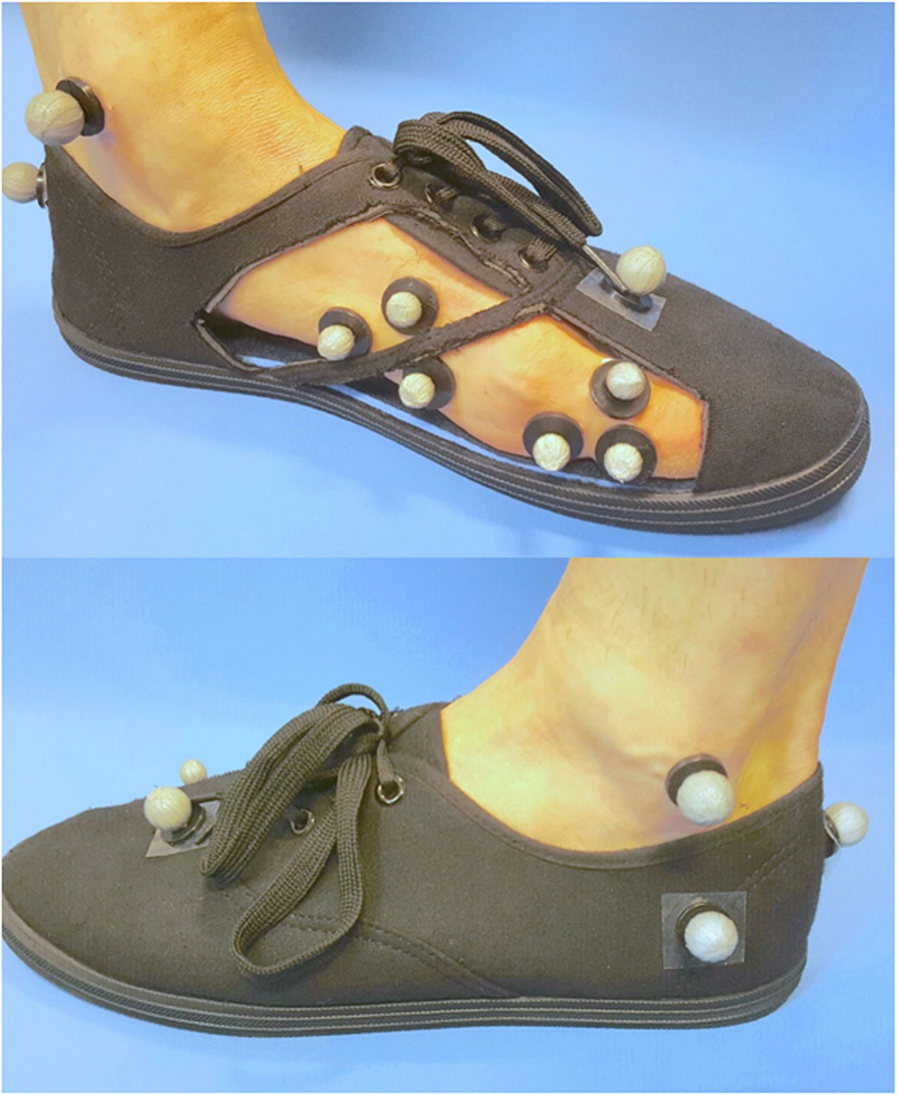
Location of foot markers used for kinematic analysis. Figure from Munteanu SE, Landorf KB, McClelland JA, Roddy E, Cicuttini FM, Shiell A, Auhl M, Allan JJ, Buldt AK, Menz HB: Shoe-stiffening inserts for first metatarsophalangeal joint osteoarthritis (the SIMPLE trial): study protocol for a randomised controlled trial. *Trials* 2017, 18:198

Biomechanical assessment was performed to evaluate sagittal plane kinematics of the 1st MTP, hip, knee and ankle joints. Kinematics were measured using a 10-camera infrared motion analysis system (Vicon Motion Systems Ltd., UK). Dorsiflexion of the hallux during walking was measured by attaching six passive retro-reflective markers to the medial forefoot (3 markers) and proximal phalanx of the hallux (3 markers) as required for calculation of 1st MTP joint kinematics using a modification of the Salford Foot Model [14]. This model has been used to assess 1st MTP joint kinematics with acceptable reliability [14]. In addition, 32 markers were fixed to anatomical landmarks of the trunk, pelvis and lower limb based on the modified Helen Hayes marker set [15, 16], as well as a customised model to allow for segmental definition and functional joint calibration. Marker trajectories were collected at a frequency of 100 Hz, and all lower limb joint kinematics were calculated based on Euler angles and described in terms of movement of the distal segment relative to the proximal segment. Data were collected and averaged from the middle stride of six 10-m walking trials for each condition at self-selected walking speed. Participants were equipped with ‘gait shoes’ with a laced fastening and canvas upper, customised with cut-outs in order to allow clear visualisation of the foot markers (Fig. 2). The minimum and maximum angles throughout the stance phase of gait were extracted from each of the six strides and averaged to represent gait for each individual. The range of motion of each joint was calculated by subtracting the minimum angle from the maximum angle.

### Statistical analysis

Statistical analysis was undertaken using SPSS version 26.0 (IBM Corp, NY, USA). We included one foot only for each participant. The symptomatic side was included (either right or left), and in the case of bilateral symptoms, the most symptomatic foot only was analysed. All data were screened for normality and outliers. Analysis was then undertaken in three stages. Firstly, associations between passive NWB 1st MTP joint maximum dorsiflexion, participant characteristics (age, height, weight, BMI and pain severity), temporo-spatial gait characteristics (velocity, cadence and step length) and the kinematic gait variables were analysed using Pearson’s *r* correlation coefficients, as these variables were considered to be possible confounders. Secondly, associations between passive NWB 1st MTP joint maximum dorsiflexion and the kinematic gait variables were analysed, and where necessary, adjusted for confounders using partial Pearson’s *r* correlation coefficients. Statistical significance was set at *p* < 0.05. Correlation coefficients were interpreted using the following cut-off values: 0 to 0.29 (weak), 0.30 to 0.49 (moderate), 0.50 to 1.00 (large) [17]. Finally, for all significant correlations, *r*^2^ values were calculated to express the proportion of variance in kinematic variables explained by 1st MTP joint maximum dorsiflexion. Sagittal plane kinematic variables that were included in the analysis were: 1st MTP joint maximum dorsiflexion, ankle joint maximum plantarflexion, ankle joint maximum dorsiflexion, ankle joint excursion, knee joint maximum extension, knee joint maximum flexion, knee joint excursion, hip joint maximum extension, hip joint maximum flexion and hip joint excursion.

## Results

### Participants

One hundred participants (45 men and 55 women, age 24 to 82 years, mean 57.5 [SD 10.3]) were recruited for the randomised trial. Of these, 54 participants were available and consented to biomechanical analysis, and complete data were available for 48 participants (24 males and 24 females). Characteristics of these participants are reported in Table 1.

**Table 1:** Participant characteristics. Values are mean (SD) unless otherwise noted

Demographics and anthropometrics	
Age – years	57.8 (10.5)
Female – n (%)	24 (50)
Height – cm	168.1 (8.3)
Weight – kg	80.0 (14.2)
Body mass index – kg/m^2^	28.4 (4.6)
Clinical features	
Passive NWB 1st MTP joint maximum dorsiflexion – mean (SD) [range], degrees^a^	45.1 (10.7) [17–62]
Pain duration – median [range], months	48.0 [6–432]
Pain on palpation – n (%)	48 (100)
Palpable dorsal exostosis – n (%)	48 (100)
Pain on motion of 1st MTP joint – n (%)	35 (72.9)
Hard-end feel when dorsiflexed – n (%)	44 (91.7)
Crepitus – n (%)	16 (33.3)
Radiographic features – n (%)^b^	
Dorsal osteophytes	44 (91.7)
Dorsal joint space narrowing	43 (89.6)
Lateral osteophytes	44 (91.7)
Lateral joint space narrowing	44 (91.7)
Radiographic 1st MTP joint OA^c^	42 (87.5)

^a^data non-normally distributed

^b^score > 0 using La Trobe Radiographic Atlas [13]

^c^at least one score of 2 for osteophytes or joint space narrowing from either view, using case definition from La Trobe Radiographic Atlas [13]

### Sagittal plane kinematics of the 1st MTP, ankle, knee and hip joints

**Fig. 3:**
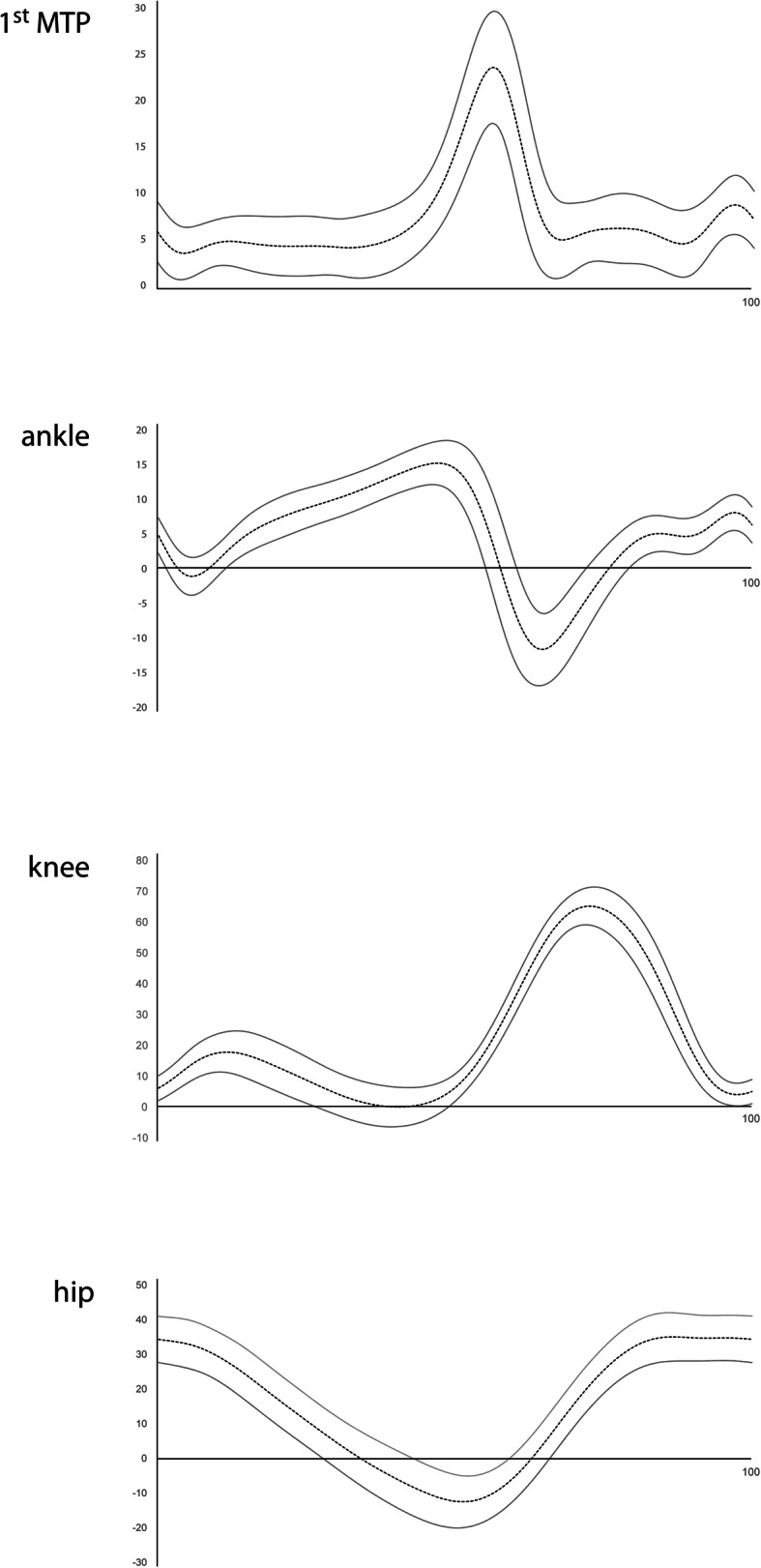
Sagittal plane kinematics (mean ± standard error, degrees) of the 1st MTP, ankle, knee and hip joints during level walking in individuals with 1st MTP joint OA. X-axis represents percentage of the gait cycle

Sagittal plane kinematics of the 1st MTP, ankle, knee and hip joints in individuals with 1st MTP joint OA are reported in Table 2 and visually presented in Fig. 3. Mean dynamic 1st MTP joint maximum dorsiflexion was 25.4 (SD 6.7) degrees.

**Table 2:** Descriptive statistics for sagittal plane kinematics (stance phase) in individuals with 1st MTP joint OA. Values are degrees

Kinematic variable	Mean (SD)	Range
1st MTP joint – maximum dorsiflexion	25.4 (6.7)	13.8 – 39.5
Ankle joint – maximum plantarflexion	7.3 (5.4)	−3.0 – –21.6
Ankle joint – maximum dorsiflexion	15.6 (3.5)	9.5–25.3
Ankle joint – total excursion	22.8 (4.4)	13.9–32.9
Knee joint – maximum extension	0.7 (5.5)	−11.7 – –11.4
Knee joint – maximum flexion	35.6 (6.5)	18.5 – 51.3
Knee joint – total excursion	36.3 (6.4)	16.1 – 51.8
Hip joint – maximum extension	13.4 (7.3)	1.6 – 27.2
Hip joint – maximum flexion	34.2 (7.1)	19.8 – 48.4
Hip joint – total excursion	47.5 (5.2)	35.1 – 57.0

### Associations between passive NWB 1st MTP joint maximum dorsiflexion and kinematic variables

**Fig. 4:**
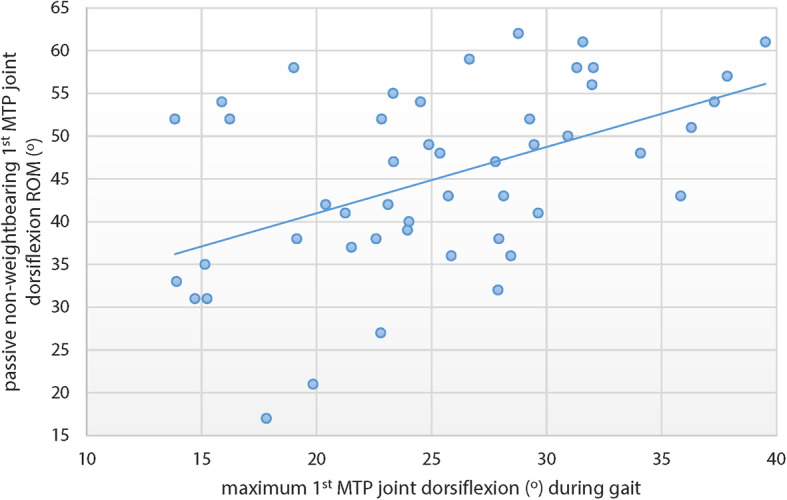
Scatterplot of correlation between passive NWB 1st MTP joint maximum dorsiflexion and 1st MTP joint maximum dorsiflexion during the stance phase of gait in individuals with 1st MTP joint OA. Pearson’s *r* = 0.486

There were no significant associations between passive NWB 1st MTP joint maximum dorsiflexion and participant characteristics (age, height, weight, BMI) or temporo-spatial gait characteristics (velocity, cadence and step length), so no adjustment for confounding was required. Associations between passive NWB 1st MTP joint maximum dorsiflexion and sagittal plane kinematics are shown in Table 3. Passive NWB 1st MTP joint maximum dorsiflexion was moderately associated with dynamic 1st MTP joint maximum dorsiflexion (*r =* 0.486, *p* < 0.01; *r*^*2*^ = 0.236), ankle joint maximum plantarflexion *r =* 0.383, *p* < 0.01; *r*^*2*^ = 0.147), and ankle joint excursion (*r =* 0.399, *p* < 0.01; *r*^*2*^ = 0.159). A scatterplot of the association between passive NWB 1st MTP joint maximum dorsiflexion and 1st MTP joint maximum dorsiflexion during stance phase is shown in Fig. 4.

**Table 3:** Associations between passive NWB 1st MTP joint maximum dorsiflexion and lower limb kinematics. Values are Pearson’s *r* correlation coefficients and *p*-values

Kinematic variable	*r*	*p*
1st MTP joint – maximum dorsiflexion	0.486	< 0.001*
Ankle joint – maximum plantarflexion	0.383	0.007*
Ankle joint – maximum dorsiflexion	−0.068	0.646
Ankle joint – excursion	0.399	0.005*
Knee joint – maximum extension	0.150	0.310
Knee joint – maximum flexion	−0.036	0.810
Knee joint – excursion	0.090	0.542
Hip joint – maximum extension	0.135	0.359
Hip joint – maximum flexion	−0.191	0.193
Hip joint – excursion	−0.067	0.652

* significant at *p* < 0.05

## Discussion

This study examined the relationship between passive NWB 1st MTP joint maximum dorsiflexion and sagittal plane kinematics in individuals with 1st MTP joint OA. Our findings indicate that individuals with less passive NWB 1st MTPJ maximum dorsiflexion exhibit less 1st MTP joint maximum dorsiflexion, less ankle joint maximum plantarflexion and ankle joint excursion during level walking. The magnitude of these associations was moderate, with *r*^*2*^ values indicating that passive NWB 1st MTP joint maximum dorsiflexion can explain approximately 24, 15 and 16% of 1st MTP joint maximum dorsiflexion, ankle joint maximum plantarflexion and ankle joint excursion, respectively. These findings are consistent with previous studies that indicate the reduction in range of motion associated with OA impairs the normal propulsive function of the foot [4, 5].

Passive NWB 1st MTP joint maximum dorsiflexion in our sample ranged from 17 to 62 degrees, with a mean of 45 degrees. Using the same measurement technique in a population-based study of 517 people aged over 50 years with foot pain, Menz et al. [2] found that passive NWB 1st MTP joint maximum dorsiflexion was associated with the radiographic severity of OA, with the most severe radiographic category demonstrating a mean value of 42 degrees. This similarity suggests that our participants were towards the more severe end of the radiographic spectrum, which would be expected given that their reported duration of OA symptoms was 4 years. 1st MTP joint maximum dorsiflexion during gait in our study ranged from 14 to 40 degrees, with a mean of 25 degrees. Despite using different kinematic models, this is similar to the mean value reported by Canesco et al. (approximately 30 degrees) in 22 patients undergoing surgery for hallux rigidus [4].

The associations reported here are consistent with Nawoczenski et al. [3] and Jarvis et al. [6], who found significant correlations between passive NWB and dynamic 1st MTP joint dorsiflexion in a pain-free healthy populations (*r =* 0.67 and *r* = 0.32, respectively). In contrast, Halstead et al. reported no significant association between passive and dynamic 1st MTP joint dorsiflexion (*r =* 0.186) [7]. However, in the Halstead et al. study, participants had limited passive 1st MTP joint maximum dorsiflexion in relaxed standing (positive Jack’s test [8]), but normal (> 50 degrees) of passive NWB 1st MTP joint maximum dorsiflexion, indicative of “functional” hallux limitus. Our study, therefore, is the first to examine this association in individuals with symptomatic, radiographically-confirmed 1st MTP joint OA.

We found a significant positive association between passive NWB 1st MTP joint maximum dorsiflexion and ankle joint maximum plantarflexion during gait, which suggests that limited 1st MTP joint dorsiflexion may impair efficient propulsion. The presence of strategies to compensate for limited 1st MTP joint dorsiflexion has been reported in previous studies, where there was an increase in lateral forefoot loading and reduced ankle joint plantarflexion in the presence of 1st MTP joint OA [5, 18, 19]. These findings have previously been linked with the high- and low-gear push-off concept proposed by Bojsen-Moller [20], whereby individuals with limited 1st MTP joint dorsiflexion fail to efficiently utilise the high-gear transverse axis (connecting the 1st and 2nd metatarsal heads) resulting in motion occurring through the low-gear oblique axis (connecting 2nd to 5th metatarsal heads) [5]. The low-gear propulsion causes a shorter lever arm between the ankle joint plantarflexors and forefoot, subsequently resulting in a higher lateral loading pattern and less efficient propulsion [5]. However, further kinematic and kinetic analyses are required to confirm this proposed mechanism.

This study has several methodological strengths, including radiographic confirmation of 1st MTP joint OA using a standardised atlas, a relatively large sample size for a kinematic study, and use of a reliable clinical measurement of passive NWB 1st MTP joint maximum dorsiflexion. However, the results of the study should be interpreted with respect to three key limitations. Firstly, due to the cross-sectional study design we cannot infer causality between passive NWB 1st MTP joint maximum dorsiflexion and kinematic changes. Secondly, kinetic data were not collected in this study, which would have allowed greater insight into the loading of the 1st MTP joint. Thirdly, our kinematic foot model was a simplified version of the Salford Foot Model [14], as participants needed to be tested while shod as part of the larger clinical trial. This precluded any analysis of the motion of the midfoot, which has been shown to be significantly altered in the presence of 1st MTP joint OA [4]. Finally, because participants were tested shod, we cannot exclude the influence of footwear on 1st MTP joint kinematics. However, the shoes used were of minimalist design with removal of large sections of the upper to accommodate the markers, so they were unlikely to have substantially influenced foot function.

In conclusion, this study identified that individuals with less passive NWB 1st MTP joint maximum dorsiflexion exhibit less dynamic 1st MTP joint maximum dorsiflexion, less ankle joint plantarflexion and less total ankle joint excursion during level walking. These findings suggest that clinical measurement of the 1st MTP joint provides useful insights into the dynamic function of the foot and ankle in this population. However, further study is required to determine the clinical importance of these observations.

## Data Availability

The datasets used and/or analysed during the current study are available from the corresponding author on reasonable request.
